# Physical and Mental Health, Technology Use, and Loneliness in Older Adults During the COVID-19 Pandemic

**DOI:** 10.1093/geroni/igab046.2237

**Published:** 2021-12-17

**Authors:** Rose Ann DiMaria-Ghalili, Martha Coates, Zachary Hathaway, Katelyn Moore, Yaegin Park, Jenny Tsui, Justine Sefcik

**Affiliations:** 1 Drexel University, College of Nursing and Health Professions, Philadelphia, Pennsylvania, United States; 2 Drexel University, Bryn Mawr, Pennsylvania, United States; 3 Drexel University, Philadelphia, Pennsylvania, United States

## Abstract

Social isolation is a negative outcome of COVID-19. This study examined patterns of physical and mental health and technology use in older adults, and loneliness during the COVID-19 pandemic. We recruited 115 community-dwelling older adults 65 and older (72% female) from the Pennsylvania region via Research Match (N=84) or from a retirement community (N=31). A significant association between loneliness and worsening of health during the pandemic was observed, Fisher’s Exact Test 6.90, p=.03. Those who were lonely demonstrated significantly lower Mental Component Summary Scores (M = 42.75, SD = 11.55) compared to those who were not lonely (M= 55.34, SD= 7.66), t(49) = 5.84, p <.01. Those reporting loneliness were more likely to use a new electronic device to communicate with family during COVID-19 pandemic, X2, (1, N= 107) = 6.24, p =.01. These findings suggest the important role of technology to decrease loneliness in older adults during a pandemic.

